# Moxifloxacin and gatifloxacin for initial therapy of tuberculosis: a meta-analysis of randomized clinical trials

**DOI:** 10.1038/emi.2016.12

**Published:** 2016-02-24

**Authors:** Qiaoling Ruan, Qihui Liu, Feng Sun, Lingyun Shao, Jialin Jin, Shenglei Yu, Jingwen Ai, Bingyan Zhang, Wenhong Zhang

**Affiliations:** Department of Infectious Diseases, Huashan Hospital, Fudan University, Shanghai 200040, China

**Keywords:** gatifloxacin, meta-analysis, moxifloxacin, randomized clinical trials, tuberculosis

## Abstract

Moxifloxacin (MOX) and gatifloxacin (GAT) have exhibited promising mycobactericidal activity, and a number of clinical trials have been conducted in recent decades to compare the treatment efficacy of MOX-containing and/or GAT-containing regimens with the standard regimen. The aim of this meta-analysis for clinical trials of MOX- or GAT-containing regimens was to evaluate their treatment efficacy and safety in initial therapy for drug-sensitive tuberculosis (TB). Databases were searched for randomized controlled trials, and nine studies with 6980 patients were included. We found that fluoroquinolone substitution for isoniazid or ethambutol in short-course regimens might result in more frequent unfavorable treatment outcomes compared with the standard regimen—in particular, an increased incidence of relapse. In a per-protocol analysis, MOX-containing regimens had slightly higher rates of sputum culture conversion at two months than the standard regimen (RR 1.08, 95% CI 1.04–1.11, *P* <0.001); there was no significant difference in the rate of sputum conversion between the GAT-containing regimens and the standard regimen (RR 1.13, 95% CI 0.96–1.33, *P =* 0.13). There were no significant differences in the incidence of death from any cause, including TB, nor were there serious adverse events between the MOX- or GAT-containing regimens and the standard regimen. In conclusion, MOX or GAT might not have the ability to shorten treatment duration in the initial therapy for tuberculosis despite the non-inferiority or even slightly better efficacy in the early phase of treatment compared with the standard regimen. Furthermore, it is safe to include MOX or GAT in initial TB treatment.

## INTRODUCTION

Tuberculosis (TB) is an infectious disease caused by *Mycobacterium tuberculosis*, and it remains a major global health problem. The World Health Organization estimated approximately 9.0 million new TB cases and 1.5 million TB-related deaths in 2013.^1^ The current recommended therapy for new cases of drug-susceptible TB is a standard 6-month regimen with four first-line drugs: isoniazid, rifampicin, ethambutol and pyrazinamide. The treatment success rate can reach up to 86% among newly diagnosed TB cases.^1^ However, the treatment duration is too long to achieve excellent compliance, which contributes to relapse and drug resistance. Therefore, new drugs or new drug combinations are required to simplify the regimen or to shorten the course of treatment with a non-inferior therapeutic effect.^2,3,4^

Both the anti-mycobacterial activity *in vitro*^5,6,7,8,9^ and the promising early sterilizing effect in animals^7,10,11,12^ led to the suggestion that fluoroquinolones (FQs) might reduce the duration of treatment in new TB cases. The fourth-generation FQs gatifloxacin (GAT) and moxifloxacin (MOX) have shown promising mycobactericidal activity,^13,14,15,16^ and a number of clinical trials have been conducted in recent decades to compare the treatment efficacy of MOX-containing and/or GAT-containing regimens with the standard regimen. In these regimens, MOX or GAT was used as the substitution for or as an addition to the established standard regimen. Previous reviews have indicated that insufficient evidence was available to assess the efficacy and safety of MOX or GAT in the first-line regimen for primary pulmonary TB and that much larger trials were required.^17,18^ A recent meta-analysis concerning the efficacy and safety of MOX plus first-line therapy failed to include relevant newly published randomized clinical trials (RCTs) and did not evaluate the relapse rates of these regimens.^19^ Given the inferiority of four-month FQ-containing regimens compared with the six-month standard regimen for drug-susceptible TB in recent large-scale trials,^20,21,22^ we speculate whether these FQ-containing regimens have sufficient anti-TB efficacy *in vivo*. Because the evidence obtained via RCTs seemed contradictory and insufficient to address this issue, we summarized the clinical trials of MOX- or GAT-containing regimens and conducted a meta-analysis to evaluate their treatment efficacy and safety in drug-susceptible TB patients.

## MATERIALS AND METHODS

### Search strategy and selection criteria

The Preferred Reporting Items for Systematic Reviews and Meta-Analyses (PRISMA)^23^ compliant literature search strategy was performed. The electronic databases MEDLINE (1966 to November 2014) and EMBASE (1974 to November 2014) were searched by two reviewers (R. and L.). We attempted to identify all relevant trials regardless of language. All of the titles and abstracts generated by this search strategy were independently reviewed. After the initial screening process, the full texts of the eligible articles were reviewed against the predefined inclusion criteria. In addition, we scrutinized the reference lists of each eligible paper for any omitted studies.

Studies were eligible for inclusion if they were RCTs that evaluated the MOX- or GAT-containing regimen for drug-sensitive pulmonary tuberculosis. Studies were excluded if they were not RCTs or if they lacked desired outcomes. Ethical approval was waived for this meta-analysis because the data we extracted were open-access from individual studies that had obtained ethics approval.

### Data extraction and outcome management

Two researchers (R. and L.) independently extracted data from studies using a pre-defined data extraction form and resolved any discrepancies in the extracted data through discussion. The basic characteristics of the studies were recorded, including author; year of publication; country; number of participants in each treatment group; mean age and gender of participants; HIV prevalence; detailed treatment regimens of each arm; and duration of follow-up.

The rate of unfavorable outcomes was set as the primary outcome. The unfavorable outcomes were treatment failure, relapse, and death or withdrawal from the trial at the end of follow-up. The rate of sputum culture conversion at the end of the intensive phase (2-month) was set as the secondary efficacy outcome. Considering the possible impact of HIV status, we had planned to assess the primary and secondary outcomes in subgroups according to HIV status, but approximately half of the studies that included HIV-infected patients did not report the relevant data, and for those that reported data according to HIV status, the outcomes of HIV-positive patients were similar to those of HIV-negative patients. We therefore did not perform a subgroup analysis. The safety outcomes were the rates of death from any cause, TB-related death and serious adverse events. We extracted the number of treatment failures, relapses, deaths, sputum culture conversions and serious adverse events, as well as the number of participants in each treatment group. We presented dichotomous data and combined them using risk ratios (RR) with 95% confidence intervals (CIs).

### Quality assessment and statistical analysis

The risk of bias for each study was assessed using the Cochrane Collaboration's tool for assessing the risk of bias.^24^ We determined whether the selected studies were appropriate for inclusion in the meta-analysis by considering six domains: sequence generation, allocation concealment, blinding (of patients, personnel and outcome assessors), incomplete outcome data, selective outcome reporting and other sources of bias (Supplementary Table). Data analysis was performed using Review Manager 5.2 (The Cochrane Collaboration, 2008; The Nordic Cochrane Centre, Copenhagen, Denmark). We assessed heterogeneity among the studies by inspecting the forest plots and by applying the χ^2^ test and inconsistency (*I*^2^) statistic.^25^ The fixed-effects model was used to calculate the RR for *I*^2^ <50%, and the random-effects model was used for *I*^2^ ≥ 50%. *P* values <0.05 suggested statistical significance.

## RESULTS

### Study selection and characteristics

**Figure 1:**
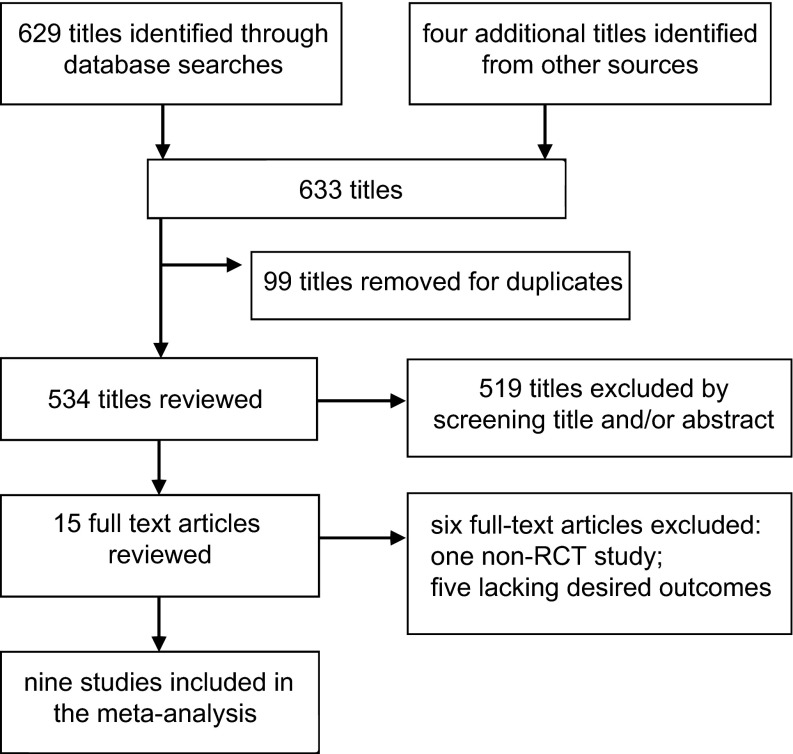
The flow diagram for the study selection

A total of 629 articles were identified from the databases, and four articles were identified from other sources. After duplicates were removed and titles and abstracts were reviewed, the full text of 15 articles was reviewed. Ultimately, nine studies met our inclusion criteria, and their data were extracted^20,21,22,26,27,28,29,30,31^ (Figure 1).

The basic characteristics of eligible RCTs are summarized in Table 1. The study locations were diverse, encompassing multiple countries. Trials were conducted mainly in Africa (Benin, Guinea, Kenya, Senegal, South Africa, Zambia, Zimbabwe, Botswana and Uganda), Asia (India, China, Malaysia and Thailand), North America (United States and Mexico), South America (Brazil) and Europe (Spain). These RCTs involved 6980 participants with a range of 170 to 1931 participants per trial. Male patients comprised 62%–74% of population, and the mean age was approximately 30 years old. Seven out of nine trials included both HIV-positive and HIV-negative patients, with the total HIV prevalence ranging from 3% to 58.5%.

Among these trials, the experimental treatment regimens could be categorized into three types: MOX/GAT as a substitute for ethambutol, MOX/GAT as a substitute for isoniazid and MOX/GAT as an addition to the standard regimen. Eight trials contained MOX in the experimental treatment regimens: five trials substituted MOX for ethambutol,^20,26,27,28,30^ three trials substituted MOX for isoniazid^20,21,29^ and one trial added MOX to the standard treatment regimen.^31^ In Gillespie's study, two MOX-containing regimens were tested in comparison with a controlled regimen.^20^ Three trials contained GAT in experimental treatment regimens, and all of these trials substituted GAT for ethambutol.^22,27,30^ All of the controlled regimens included standard doses of isoniazid, rifampicin, pyrazinamide and ethambutol. Notably, five of the nine studies used shortened regimens of four months in the experimental groups compared with the standard regimen of six months. The mean duration of follow-up ranged from two months of treatment to 24 months after the end of treatment.

### Study quality

**Figure 2:**
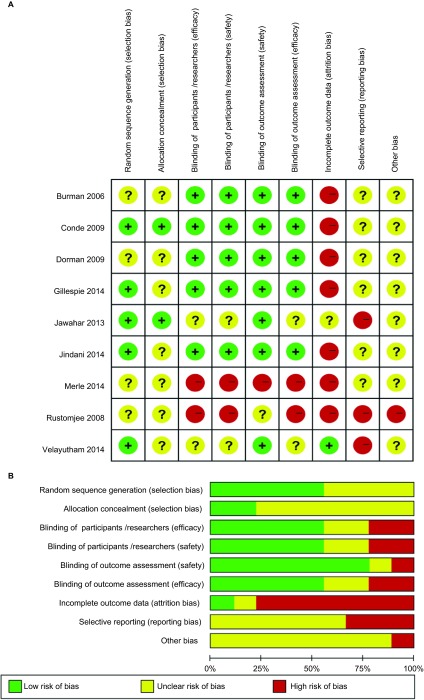
The quality assessment of each included study is summarized in (A) ‘risk of bias summary', or is presented as a percentage across all included studies in (B) ‘risk of bias graph'

According to the Cochrane methodology, the risk of bias of the included studies was assessed as summarized in Figure 2A–2B. Five trials were judged to be at low risk considering the detailed information regarding the generation of randomized sequence, whereas the remaining trials were judged to be unclear. Most studies had an unclear risk of allocation concealment in that no evidence could be found in their methodology. Five trials used blinding and a placebo and were judged to pose a low risk of bias. Seven trials were at a high risk of bias due to incomplete outcome data stemming from moderate dropout with the potential to alter results. Due to insufficient information regarding reporting and other sources of bias, most trials were judged to be of unclear risk.

### Treatment outcome

#### Unfavorable outcomes

We aimed to use the rate of unfavorable outcomes at the end of follow-up as the primary efficacy outcome. After extracting data from the included studies, we found that four studies provided data regarding the desired outcome. However, due to differences in the duration of treatment and follow-up among the studies, we could not calculate a pooled estimate. All of the relevant information is summarized in Table 2.

Three studies examined the efficacy of short-course MOX-containing regimens, and all of the unfavorable outcome rates for these 4-month regimens were higher than those of the standard regimen.^20,21,30^ Similarly, higher rates of unfavorable outcomes were obtained for short-course GAT-containing regimens compared to the standard regimen.^22,30^ Considering that treatment failure rates and relapse rates were important components of assessing treatment efficacy, we also summarize detailed data in Table 2. For treatment failures at the end of treatment, a pooled estimate was performed, and we found that there was no difference between the standard regimen and short-course regimens (RR = 0.73, 95% CI: 0.34–1.56, *P* value 0.42) (Supplementary Figure 1). In contrast, the shortened regimens led to an increased incidence of relapse during follow-up, although the data were not pooled by variation in follow-up duration among these studies. The relapse rate following the 4-month MOX-containing regimens were approximately twice as high as that of the standard regimen in Gillespie's study and Jindani's study.^20,21^ Similarly, the short-course GAT-containing regimen resulted in a higher relapse rate compared to the standard regimen (RR = 2.01, 95% CI 1.44–2.79).^22^

We aimed to conduct subgroup analysis according to HIV status to measure the possible impact of HIV co-infection. Among the seven studies that enrolled patients who were co-infected with HIV, only four reported detailed data. Two MOX-containing studies^20,21^ and one GAT-containing study^22^ compared the unfavorable outcome rate between HIV-positive patients and HIV-negative patients, and another MOX-containing study^26^ set the two-month sputum conversion rate as the end point. We therefore did not conduct this subgroup analysis. However, all seven of these studies reported no difference in effect based on HIV status.

#### Sputum conversion at two months

The rate of sputum conversion was reported in each study included in our analysis and was used as the primary efficacy point in several studies. In this meta-analysis, the pooled rates of sputum culture conversion at two months of treatment were calculated as a secondary outcome. Because a considerable number of randomized patients failed to meet the inclusion criteria, were lost to follow-up, or withdrew, a per-protocol analysis was conducted.

**Figure 3:**
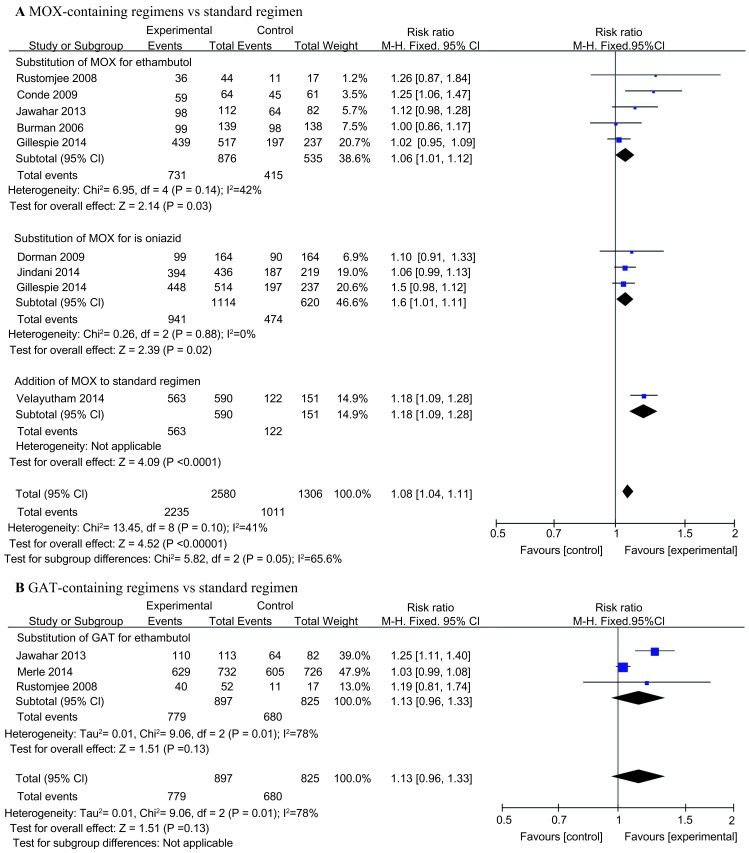
Forest plots comparing the rates of sputum conversion at two months for (A) moxifloxacin-containing regimens versus the standard regimen, and (B) gatifloxacin-containing regimens versus the standard regimen. MOX, moxifloxacin; GAT, gatifloxacin

In the per-protocol analysis, we found that MOX-containing regimens resulted in higher sputum culture conversion at two months [2235 (86.6%) of 2580 patients] than did the standard regimen [1011 (84.3%) of 1306 patients] with statistical significance (RR = 1.08, 95% CI: 1.04–1.11, *P* value <0.00001) (Figure 3A). A similar effect was observed via subgroup analysis when MOX was substituted for ethambutol or isoniazid in the experimental treatment regimens. In addition, most of the studies included did not show a significant difference. In Velayutham's study, MOX was added to the standard regimen, and the five-drug daily regimen resulted in significantly higher sputum culture conversion after the first two months compared with the standard four-drug regimen (RR = 1.18, 95% CI 1.09–1.28,*P* value <0.001).^31^ We detected moderate heterogeneity, with *I*^2^ = 41%. In addition, solid culture medium seemed to produce more negative culture results than liquid culture. In the per-protocol analysis of data from solid culture results, we found that MOX-containing regimens resulted in a much higher sputum culture conversion rate at two months [1934 (90.0%) of 2148 patients] than did the standard regimen [800 (78.1%) of 1024 patients] with statistical significance (RR = 1.16, 95% CI: 1.05–1.28, *P* value <0.00001) (Supplementary Figure 2). However, the data for liquid culture were heretofore insufficient.

For GAT-containing regimens, the pooled rate of sputum conversion at two months of the experimental groups [779 (86.8%) of 897 patients] was slightly higher than that in the control groups [680 (82.4%) of 825 patients], but no significant difference was found (RR: 1.13, 95% CI: 0.96–1.33, *P* value = 0.13) (Figure 3B). However, heterogeneity was found between these studies, with *I^2^* = 78%.

#### Safety outcomes

**Figure 4:**
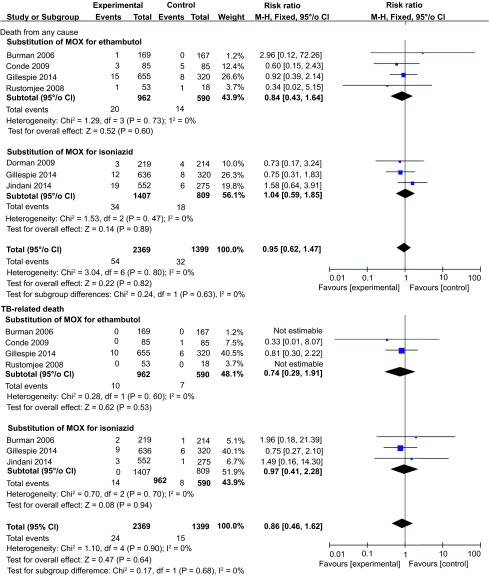
Forest plots comparing the rates of death from any cause and TB-related deaths between MOX-containing regimens and the standard regimen. MOX, moxifloxacin

**Figure 5:**
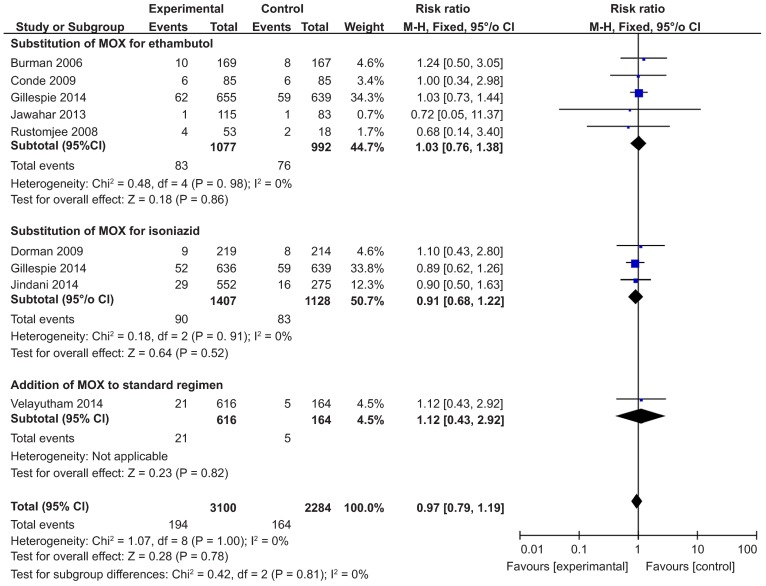
A forest plot comparing the rates of serious adverse events between MOX-containing regimens and the standard regimen. MOX, moxifloxacin

We also performed an analysis of safety outcomes and found no difference in the rates of death from any cause, TB-related death, or serious adverse events between MOX-containing regimens and the standard regimen (Figure 4 and 5). No heterogeneity was found, and the *I^2^* value was very low. As with MOX, there was no significant difference in the number of serious adverse events between GAT-containing regimens and the standard regimen (Table 3). Two trials reported data on the number of deaths from any cause as well as TB-related deaths and found no significant difference between the experimental and control groups.^22,27^

## DISCUSSION

In the present study, we summarized the efficacy and safety of MOX and GAT as a part of first-line regimens for the treatment of drug-sensitive TB cases. Nine RCTs were eligible for our meta-analysis, and a total of 6980 participants were included. Our results indicate that FQ substitution for isoniazid or ethambutol in short-course regimens resulted in more frequent unfavorable treatment outcomes compared with the standard regimen, especially in terms of relapse. The MOX-containing regimens had a slightly higher sputum culture conversion rate at two months than did the standard regimen, but the GAT-containing regimens failed to achieve a similar result. There were no significant differences in the incidence of death from any cause, in TB-related death, or in serious adverse events between the MOX- or GAT-containing regimens and the standard regimen.

MOX, as is the case with isoniazid, has greater early bactericidal activity than other standard drugs. Previous studies have indicated that MOX greatly accelerated the speed of culture conversion and produced a stable cure when combined with rifampin and pyrazinamide in a mouse model.^11,12^ It also exhibited early bactericidal activity that was comparable with that of isoniazid in TB patients.^15^ In this meta-analysis, we found a slight increase in the sputum culture conversion rate at two months when either ethambutol or isoniazid was replaced by MOX. The equivalence of the sputum conversion results may suggest an important role of MOX in the treatment of TB, particularly in patients intolerant to isoniazid or carrying isoniazid-resistant strains. However, the highly significant differences in sputum conversion (*P* <0.00001) corresponded to only slight differences in the conversion rates (86.6% *vs.* 84.3%). Of the phase two trials included in our meta-analysis, most investigators used 2-month sputum culture conversions as their primary efficacy endpoint, which is a surrogate marker for the final treatment outcome. Two-month culture status has been demonstrated in earlier studies to be a good marker for the efficacy of TB treatment regimen, but the observed correlation among populations is not strong enough to reliably predict treatment outcomes such as relapse. Based on the detailed data provided in Table 2, it seems that the 6-month MOX-containing regimen resulted in similar relapse rates compared to the standard regimen used in Jindani's study,^21^ whereas a higher relapse rate was reported when the regimens were shortened to 4 months in the three phase three clinical trials.^20–22^ The higher relapse rate indicated that MOX or GAT substitution might not permit a reduction in treatment duration. This result could be influenced by the shorter therapeutic period. In future studies, more robust surrogate markers of treatment efficacy are needed to select suitable regimens for shortening tuberculosis treatment that can be further assessed in phase three studies. Interestingly, Wallis and his colleagues developed a meta-regression model in 2013 to predict relapse risk using treatment duration and the 2-month sputum culture positive rate as predictors, and the analysis using this model of published2-month data for MOX-containing regimens indicated that it would result in relapse rates similar to those of standard therapy only if administered for over five months.^32,33^ They further assessed and refined the model in 2015 using more recent RCTs. This finding may provide information regarding treatment duration for future TB trials. Regretfully, a number of RCTs comparing non-shortened FQ-containing regimens with the standard regimen lacked data describing relapses and deaths due to an insufficient duration of follow-up.^26,27,28,29^ Thus, we could not determine whether non-shortened FQ-containing regimens resulted in better outcomes than the standard regimen. One study indicated that supplementing the controlled regimen with MOX could significantly hasten culture conversion.^31^ However, this study was an interim analysis, and research regarding the rate of treatment success and relapse is in progress. Whether adding MOX or GAT to the standard regimen successfully shortens the initial treatment regimen remains unknown, and more studies are needed. Furthermore, the contradictory outcomes of animal studies and human trials indicate that current available mouse models do not fully recapitulate human TB infection; caution should be exercised when interpreting these studies.

In the safety analysis, there were no significant differences in the incidence of death from any causes, TB-related deaths between FQ-containing regimens and the standard regimen. Adverse events were recorded in each study, especially for certain symptoms: GAT had been reported to cause incidents of hypoglycemia and hyperglycemia,^34^ and MOX was shown to prolong the QTc interval.^35^ Our meta-analysis revealed that there were no significant differences between the groups regarding the percentage of serious adverse events that were considered relevant to the studied medications. However, studies of the use of GAT to treat TB were insufficient compared to those for MOX. Table 3 shows the rates of death (from TB or from unrelated causes) and of serious side effects from three studies of GAT. However, one of these studies did not provide information regarding deaths, and another had a follow-up of only two months, rendering them insufficient to evaluate long-term safety. Thus, a precise safety assessment of GAT-containing regimens requires more study.

Furthermore, the use of FQs for initial tuberculosis therapy raises concerns regarding whether the application of FQs in drug-sensitive TB will increase the rate of FQ-resistant *M. tuberculosis* isolates, thereby hampering the treatment of multi-drug resistant TB (MDR-TB) in more difficult situations. FQs currently play a very important role in the treatment of MDR-TB^18,36^ and are strongly recommended by the WHO guidelines for the programmatic management of drug-resistant tuberculosis.^1,37^ They are significantly associated with cure—an effect that is more pronounced for later-generation FQs, such as levofloxacin, MOX and GAT.^37^ However, several studies have reported a high prevalence of FQ-resistant TB in China,^38^ India^39^ and the Philippines.^40^ In Migliori's meta-analysis, TB patients were found to have a three-fold higher risk of acquiring FQ-resistant TB when prescribed FQs before TB diagnosis compared to non-FQ-exposed patients (OR 2.81, 95% CI 1.47–5.39).^41^ In another meta-analysis, previous exposure to FQs was determined to be a risk factor for FQ-resistant TB; it was found that 20.8% of patients exposed to FQs for more than 10 days, 60 days prior to a TB diagnosis had FQ-resistant TB compared to only 1.6% of those taking an FQ for less than 10 days.^42^ Thus, if we use MOX for initial therapy, it is possible that FQ-resistant MDR-TB strains will flourish, leading to the development of extensively drug-resistant tuberculosis.

This meta-analysis has several limitations. First, variation in treatment duration and the follow-up period among the studies resulted in the inability to calculate a pooled estimate of the rate of unfavorable outcomes. Studies that use FQ as a component in the first-line non-shortened regimen with an adequate follow-up duration are needed to fully evaluate the efficacy of FQs. Second, although culture conversion is the most widely supported surrogate endpoint, the discrepancy between sputum culture conversion rate at two months and treatment outcome indicated that the relapse rate after the completion of therapy could be a precise indicator of effectiveness. However, among the nine included studies, only four studies reported the number of relapses. Third, only a handful of studies using GAT to treat TB were identified, and the duration of treatment and follow-up varied, which contributed to the moderate heterogeneity when we calculated the pooled estimates. Thus, the calculation of the rates of death and adverse events between GAT-containing regimens and the standard regimen may be crude, and the precise efficacy and safety of GAT in initial therapy of TB requires additional study. Fourth, although pooling the results using different culture methods may result in greater heterogeneity, the results should not impact the main finding.

In conclusion, MOX or GAT might not be able to shorten treatment duration in the initial therapy for TB, despite their equivalent or even slightly better efficacy in early phase of treatment compared with the standard regimen. Nevertheless, it is safe to include MOX or GAT in initial TB treatment.

## Figures and Tables

**Table 1:** Basic characteristics of the studies included in this meta-analysis

**Study**	**Country**	**Enrolled patients**	**Number of each arm^a^**	**Male**	**Mean age (interquartile range)**	**HIV prevalence**	**Treatment regimens^b^**		**Duration of follow-up**
							**Experimental group**	**Control group**	
Burman *et al.*^26^	Africa, North America	336	169 vs 167	67%	31 (24–40)	21.7%	2HRZM/4HR	2HRZE/4HR	2 m
Rustomjee *et al.*^27^	South Africa	217	163 vs 54	66.8%	31.5 (25–37)	58.5%	2HRZM/4HR2HRZG/4HR2HRZO/4HR	2HRZE/4HR	2 m
Dorman *et al.*^29^	North America, Brazil, South Africa, Spain, Uganda	433	185 vs 196	72%	30 (25–38)	11%	2RZEM/4HR	2HRZE/4HR	2 m
Conde *et al.*^28^	Brazil	170	74 vs 72	62%	32 (na)	3%	2HRZM/4HR	2HRZE/4HR	18 m
Jawahar *et al.*^30^	South India	429	251 vs 165	74%	na	0	2HRZM/2HRM2HRZG/2HRG	2HRZE/4HR	30 m
Velayutham *et al.*^31^	South India	801	616 vs 164	75%	na	0	3HRZEM;2HRZEM/2HRM(daily);2HRZEM/2HRM(thrice wk)	2HRZE/4HR(thrice wk)	2 m
Jindani *et al.*^21^	South Africa, Zimbabwe, Botswana, Zambia	827	552 vs 275	64%	na	27%	2R(high-dose)ZEM/2MP2R(high-dose)ZEM/4MP	2HRZE/4HR	18 m
Gillespie *et al.*^20^	South Africa, India, Kenya, Thailand, Malaysia, Zambia, China, Mexico	1931	1291vs 640	70%	na	7%	4HRZM4ERZM	2HRZE/4HR	
Merle *et al.*^22^	Benin, Guinea, Kenya, Senegal, South Africa	1836	848 vs 844	72.7%	30.7(na)	18.1%	2HRZG/2HRG	2HRZE/4HR	30 m

H, isoniazid; R, rifampicin; E, ethambutol; Z, pyrazinamide; M, moxifloxacin; G, gatifloxacin; O, ofloxacin; P, rifapentine; na, not available.

a Number of patients in an experimental group vs a control group in intention-to-treat analysis.

b In each regimen, the number indicates the number of months of treatment: e.g., ‘2HRZM/4HR' represents two months of treatment with HRZM followed by four months of treatment with HR.

**Table 2:** Summary of the rates of unfavorable outcomes, including treatment failure and relapse, between MOX-/GAT-containing regimens and the standard regimen in an intention-to-treat analysis or a modified intention-to-treat analysis

**Study**	**Treatment regimens^a^**	**Unfavorable outcome rates**	**Treatment failure rates^b^**	**Relapse rates^b^**				
	**Experimental**	**Control**	**Experimental**	**Control**	**Experimental**	**Control**	**Experimental**	**Control**
Jawahar *et al.*^30^	2HRZM/2HRM	2HRZE/4HR	13.9%	8.1%	2/108 (1.9%)	2/137 (1.5%)	11/104 (10.6%)^c^	8/132 (6.1%)^c^
	2HRZG/2HRG		16.9%		6/118 (5.1%)		17/115 (14.8%)^c^	
Merle *et al.*^22^	2HRZG/2HRG	2HRZE/4HR	21%	17.2%	12/694 (1.4%)	16/662 (2.4%)	101/694 (14.6%)^d^	47/662 (7.1%)^d^
Gillespie *et al.*^20^	4HRZM	2HRZE/4HR	23%	16%	5/568 (0.9%)	7/555 (1.3%)	46/568 (8.1%)	13/555 (2.3%)
	4ERZM		24%		5/551 (0.9%)		64/551 (11.6%)	
Jindani *et al.*^21^	2R(high-dose)ZEM/2MP	2HRZE/4HR	26.9%	14.4%	2/193 (1.0%)	2/188 (1.1%)	27/193 (15.8%)	6/188 (3.1%)
	2R(high-dose)ZEM/4MP		13.7%		0/212 (0.0%)		5/212 (2.7%)	

H, isoniazid; R, rifampicin; E, ethambutol; Z, pyrazinamide; M, moxifloxacin; G, gatifloxacin; P, rifapentine; na, not available.

a In each regimen, the number indicates the number of months of treatment: e.g., ‘2HRZM/4HR' represents two months of treatment with HRZM followed by four months of treatment with HR.

b The number of patients experiencing treatment failure or relapse during the follow-up/the number of patients included in each arm (rate).

c Jawahar's study reported the rates of recurrence during the follow-up period in the absence of genotyping, but the authors speculated that these recurrences were likely to be relapses rather than re-infections.

d In Merle's study, the recurrence rates were provided. The relapse rates were not available because only 55% of strains from patients with a culture-positive recurrence were genotyped to distinguish relapse from re-infection. Among these genotyped strains, 75% of patients in the experimental group and 81% of the patients in the control group had a relapse.

**Table 3:** Summary of the safety outcomes of the GAT-containing regimens and the standard regimen in the included studies

**Study**	**Experimental regimen^a^**	**Control regimen^a^**	**Follow-up (month)**	**Death from any cause^b^**	**TB-related death^b^**	**Serious side effects^b^**			
				**Experimental**	**Control**	**Experimental**	**Control**	**Experimental**	**Control**
Rustomjee *et al.*^27^	2HRZG/4HR	2HRZE/4HR	2	0/55 (0%)	2/54 (3.7%)	0/55 (0%)	0/50 (0%)	3/55 (5.5%)	7/54 (13.0%)
Jawahar *et al.*^30^	2HRZG/2HRG	2HRZE/4HR	24	na	na	na	na	4/136 (2.9%)	1/165 (0.6%)
Merle *et al.*^22^	2HRZG/2HRG	2HRZE/4HR	30	10/848 (1.2%)	12/844 (1.4%)	2/848 (0.2%)	3/844 (0.4%)	20/848 (2.4%)	23/844 (2.7%)

H=isoniazid, R=rifampicin, E=ethambutol, Z=pyrazinamide, M=moxifloxacin, G=gatifloxacin. na=not available.

a In each regimen, the number indicates the number ofmonths of treatment: e.g., “2HRZG/4HR” represents twomonths of treatment with HRZG followed by four months of treatment with HR.

b The number of events or the number of patients included in each arm (rate).
