# Supplementary material for: Moxifloxacin and gatifloxacin for initial therapy of tuberculosis: a meta-analysis of randomized clinical trials
Source: Emerg Microbes Infect. 2016 Feb 24;5(2):e12–. doi: 10.1038/emi.2016.12 (PMC4777926; doi:10.1038/emi.2016.12)
Supplement: Supplementary Information [file emi201612x1.docx]

**Supplementary table** Summary of the support for the judgment of the risk of bias among included studies.

Burman 2006

| **Bias** | **Author’s judgment** | **Support for judgment** |
| --- | --- | --- |
| Random sequence generation | Unclear risk | “randomized in a factorial design”; “Randomization was stratified by continent of enrolment and presence of pulmonary cavitations” |
| Allocation concealment | Unclear risk | Method of concealment not described. |
| Blinding of participants/researchers(efficacy) | Low risk | Not described in study report. However the trial was double-dummy placebo controlled. Review authors judged that the efficacy outcomes were not likely to be influenced by lack of blinding |
| Blinding of participants/researchers(safety) | Low risk |  |
| Blinding of outcome assessment(efficacy) | Low risk |  |
| Blinding of outcome assessment(safety) | Low risk |  |
| Incomplete outcome data | High risk | 59/336 (17.6%) excluded from final analysis. |
| Selective reporting | Unclear risk | Insufficient information to permit judgment |
| Other bias | Unclear risk | Two of 12 authors had a financial conflict of interest. Bayer Pharmaceuticals donated moxifloxacin and moxifloxacin placebo tablets |

Conde 2009

| **Bias** | **Author’s judgment** | **Support for judgment** |
| --- | --- | --- |
| Random sequence generation | Low risk | “permuted block randomization with blocks of four” |
| Allocation concealment | Low risk | “allocation slips sealed in opaque envelopes opened after enrolment” |
| Blinding of participants/researchers(efficacy) | Low risk | Double dummy placebo control was used. Patients, study clinicians, and study staff were unaware of the treatment assignments of patients with the exception of the pharmacist who dispensed medication packets |
| Blinding of participants/researchers(safety) | Low risk |  |
| Blinding of outcome assessment(efficacy) | Low risk |  |
| Blinding of outcome assessment(safety) | Low risk |  |
| Incomplete outcome data | High risk | 45/170 (26.5%) excluded from final analysis. |
| Selective reporting | Unclear risk | Insufficient information to permit judgment |
| Other bias | Unclear risk | The authors declared no conflict of interest. Bayer Healthcare donated moxifloxacin and matching placebo, but had no input into the study design, execution, or analysis. Authors described the role of the funding source in the trial from design to publication of report |

Dorman 2009

| **Bias** | **Author’s judgment** | **Support for judgment** |
| --- | --- | --- |
| Random sequence generation | Unclear risk | Study report specified “randomly assigned” but did not mention the method of randomization. |
| Allocation concealment | Unclear risk | Not described. |
| Blinding of participants/researchers(efficacy) | Low risk | Double dummy placebo control was used. Review authors judged that safety outcomes were unlikely to have been influenced by a lack of blinding |
| Blinding of participants/researchers(safety) | Low risk |  |
| Blinding of outcome assessment(efficacy) | Low risk |  |
| Blinding of outcome assessment(safety) | Low risk |  |
| Incomplete outcome data | High risk | 105/433 (24.3%) excluded from final analysis. |
| Selective reporting | Unclear risk | Insufficient information to permit judgment. |
| Other bias | Unclear risk | Three out of 19 authors had a financial conflict of interest. Bayer Pharmaceuticals provided moxifloxacin and moxifloxacin placebo tablets. |

Gillespie 2014

| **Bias** | **Author’s judgment** | **Support for judgment** |
| --- | --- | --- |
| Random sequence generation | Low risk | During randomization, patients were assigned a unique study number selected sequentially from the appropriate randomization list that corresponded to the treatment pack allocated. |
| Allocation concealment | Unclear risk | Not described. |
| Blinding of participants/researchers(efficacy) | Low risk | double-blind, placebo-controlled, Only statisticians who were responsible for preparing the reports for the independent data and safety monitoring committee and essential manufacturing and distribution staff members had access to the list of identifiers matched to the intervention. |
| Blinding of participants/researchers(safety) | Low risk |  |
| Blinding of outcome assessment(efficacy) | Low risk |  |
| Blinding of outcome assessment(safety) | Low risk |  |
| Incomplete outcome data | High risk | 19.8%（383/1931）excluded from final analysis. |
| Selective reporting | Unclear risk | Insufficient information to permit judgment. |
| Other bias | Unclear risk | The authors declared no conflict of interest. Bayer Healthcare for the donation of moxifloxacin; and Sanofi for the donation of rifampin. |

Jawarha 2013

| **Bias** | **Author’s judgment** | **Support for judgment** |
| --- | --- | --- |
| Random sequence generation | Low risk | “Restricted random allocation sequences were generated by a biostatistician using random number tables, separately for the two strata and sealed.“ |
| Allocation concealment | Low risk |  |
| Blinding of participants/researchers(efficacy) | Unclear risk | Study report gave no information on this part of blinding. |
| Blinding of participants/researchers(safety) | Unclear risk |  |
| Blinding of outcome assessment(efficacy) | Low risk | “Bacteriological investigations were carried out by technicians who were blinded to the clinical status of the patient and the regimen.” |
| Blinding of outcome assessment(safety) | Unclear risk | Study report gave no information on this part of blinding. |
| Incomplete outcome data | Unclear risk | 7/403 were excluded from final analysis. “On review of interim data, the Data and Safety Monitoring Board (DSMB) recommended termination of the G arm initially (February 2006), and later the M arm (October 2006) due to high TB recurrence rates in these two arms compared to the control regimen arm.” |
| Selective reporting | High risk | No data and estimates on serious adverse events. |
| Other bias | Unclear risk | The authors declared no conflict of interest. Bayer Healthcare for the donation of moxifloxacin; and Sanofi for the donation of rifampin. |

Jindani 2014

| **Bias** | **Author’s judgment** | **Support for judgment** |
| --- | --- | --- |
| Random sequence generation | Low risk | “A randomized allocation sequence was generated for each study center with the use of blocks of varying size by an independent statistician based at the MRC CTU.” |
| Allocation concealment | Unclear risk | Not described. |
| Blinding of participants/researchers(efficacy) | Low risk | Apart from the statisticians reporting to the data and safety monitoring committee,the staff at St. George’s and at the MRC CTU were unaware of treatment assignment except when a lack of awareness would have been unethical (e.g., in some discussions of seriousadverse events). Participating laboratories wereunaware of treatment assignment throughout the study. |
| Blinding of participants/researchers(safety) | Low risk |  |
| Blinding of outcome assessment(efficacy) | Low risk |  |
| Blinding of outcome assessment(safety) | Low risk |  |
| Incomplete outcome data | High risk | 234/827 were excluded from final analysis. |
| Selective reporting | Unclear risk | Insufficient information to permit judgment. |
| Other bias | Unclear risk | No potential conflict of interest relevant to this article was reported. |

Merle 2014

| **Bias** | **Author’s judgment** | **Support for judgment** |
| --- | --- | --- |
| Random sequence generation | Unclear risk | “Patients were randomly assigned, in a 1:1 ratio with stratification according to country.” |
| Allocation concealment | Unclear risk | Not described. |
| Blinding of participants/researchers(efficacy) | High risk | Study report gave no information on blinding but study was open-labeled. |
| Blinding of participants/researchers(safety) | High risk |  |
| Blinding of outcome assessment(efficacy) | High risk |  |
| Blinding of outcome assessment(safety) | High risk |  |
| Incomplete outcome data | High risk | 26%(1692-1256/1692)were excluded in the final analysis. |
| Selective reporting | Unclear risk | Insufficient information to permit judgment. |
| Other bias | Unclear risk | The authors declared no conflict of interest.Lupin Pharmaceuticals had no role in the conduct of the trial, the analysis of the data, or the preparation of the manuscript |

Rustomjee 2008

| **Bias** | **Author’s judgment** | **Support for judgment** |
| --- | --- | --- |
| Random sequence generation | Unclear risk | “Patients were randomly allocated in successive blocks of 20 equally to one of four regimens for the first 8 weeks of treatment.” |
| Allocation concealment | Unclear risk | Not described. |
| Blinding of participants/researchers(efficacy) | High risk | Study was open-labeled. |
| Blinding of participants/researchers(safety) | High risk |  |
| Blinding of outcome assessment(efficacy) | Unclear risk | Study was open-labeled but laboratory staff were likely blinded to treatment assignment. Review authors judged that bacteriological efficacy outcomes were unlikely to have been at high risk of bias. |
| Blinding of outcome assessment(safety) | High risk | Study was open-labeled. |
| Incomplete outcome data | High risk | 18 participants (8.3%) excluded. |
| Selective reporting | High risk | Authors did not present data on the most frequent adverse events by study group or on cause of death by study group, or time of death. |
| Other bias | High risk | No conflict of interest statement. |

Velayutham 2014

| **Bias** | **Author’s judgment** | **Support for judgment** |
| --- | --- | --- |
| Random sequence generation | Low risk | Allocation sequences generated using random number tables, separately for the 6 strata, were used to assign the regimens. |
| Allocation concealment | Unclear risk | Not described. |
| Blinding of participants/researchers(efficacy) | Unclear risk | No information about blinding. No placebo. |
| Blinding of participants/researchers(safety) | Unclear risk |  |
| Blinding of outcome assessment(efficacy) | Low risk | Bacteriological examinations were carried out in a blinded fashion and technicians were unaware of the clinical status of the patient and the regimen. |
| Blinding of outcome assessment(safety) | High risk | No information about blinding. |
| Incomplete outcome data | Low risk | 39/780 were excluded from the final analysis. |
| Selective reporting | High risk | No details about death cause nor the amount of serious adverse events. |
| Other bias | Unclear risk | No reported conflicts. |
