# Supplementary figures and images for: Moxifloxacin and gatifloxacin for initial therapy of tuberculosis: a meta-analysis of randomized clinical trials
Source: Emerg Microbes Infect. 2016 Feb 24;5(2):e12–. doi: 10.1038/emi.2016.12 (PMC4777926; doi:10.1038/emi.2016.12)

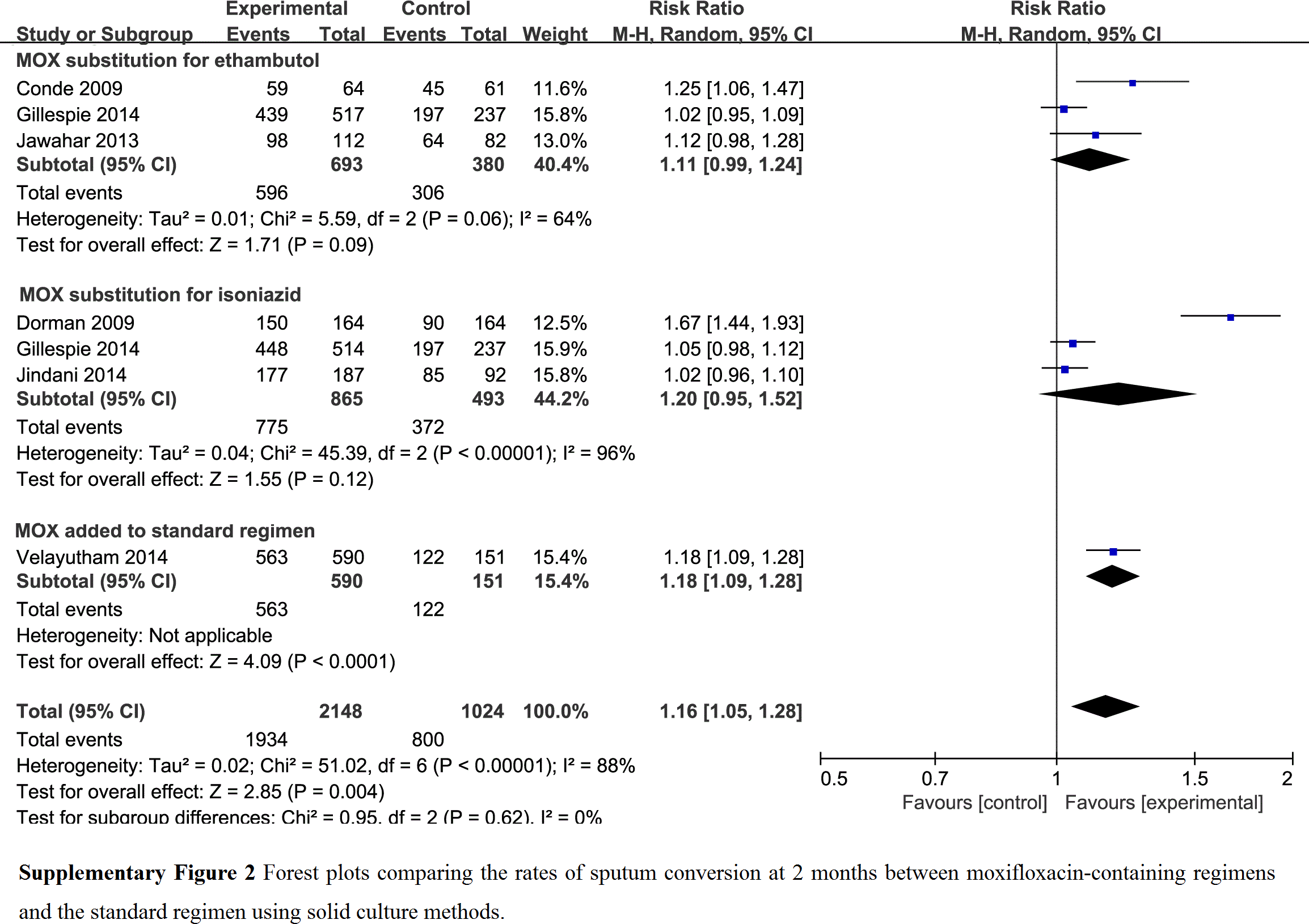

Supplement: Supplementary Information [file emi201612x3.tif]
